# 

**Published:** 1920-03

**Authors:** 


					CONTENTS.   Page
Queene Elizabethes Achademy.
By L. M. Griffiths, M.R.C.S. Eng.,
L.R.C.P. Ed.
0nrl? Considerations on the Applications of Physiology to
Medicine.
By G. A. Buckmaster, M.A., M.D. Oxon.
The Modern Treatment of Tuberculosis of the Spine.
By
a. kendle Short, M.D., B.S., B Sc., F.R.C.S.
19
0 ?a^CS ^ncePhalitis Lethargica in Bristol.
By D. S. Davies,
J. O. Symes, F. H. Edgeworth, I. Walker IIall and J. A. Nixon
25
* Malaria.
A Discussion by D. S. Davies, S. Kerfoot and Cecil Clarke
38
Case of Lymphosarcoma of the Tonsil and Palate.
By P. Watson-
Villiams, M.D. Lond.
5i
progress of the Medical Sciences :
Medicine.
J- A. Nixon, M.D. Cantab. ..
52
Surgery.
James Swain, M.D., M.S. Lond.
55
; Reviews of Bookj
59
,Editorial Notes
73
KThe Library of the Bristol Medico-Chirurgical Society ... ... 76
Meetings of Societies ...
78
Local Medical Notes
79

				

